# The Post-Translational Regulation of Epithelial–Mesenchymal Transition-Inducing Transcription Factors in Cancer Metastasis

**DOI:** 10.3390/ijms22073591

**Published:** 2021-03-30

**Authors:** Eunjeong Kang, Jihye Seo, Haelim Yoon, Sayeon Cho

**Affiliations:** Laboratory of Molecular and Pharmacological Cell Biology, College of Pharmacy, Chung-Ang University, Seoul 06974, Korea; ejaykang@gmail.com (E.K.); seojh0228@gmail.com (J.S.); limtiny@naver.com (H.Y.)

**Keywords:** metastasis, epithelial–mesenchymal transition, transcription factor, Snail, Twist, ZEB

## Abstract

Epithelial–mesenchymal transition (EMT) is generally observed in normal embryogenesis and wound healing. However, this process can occur in cancer cells and lead to metastasis. The contribution of EMT in both development and pathology has been studied widely. This transition requires the up- and down-regulation of specific proteins, both of which are regulated by EMT-inducing transcription factors (EMT-TFs), mainly represented by the families of Snail, Twist, and ZEB proteins. This review highlights the roles of key EMT-TFs and their post-translational regulation in cancer metastasis.

## 1. Introduction

Morphological alteration in tissues is related to phenotypic changes in cells [1]. Changes in morphology and functions of cells can be caused by changes in transcriptional programs and protein expression [2]. One such change is epithelial–mesenchymal transition (EMT).

**Figure 1:**
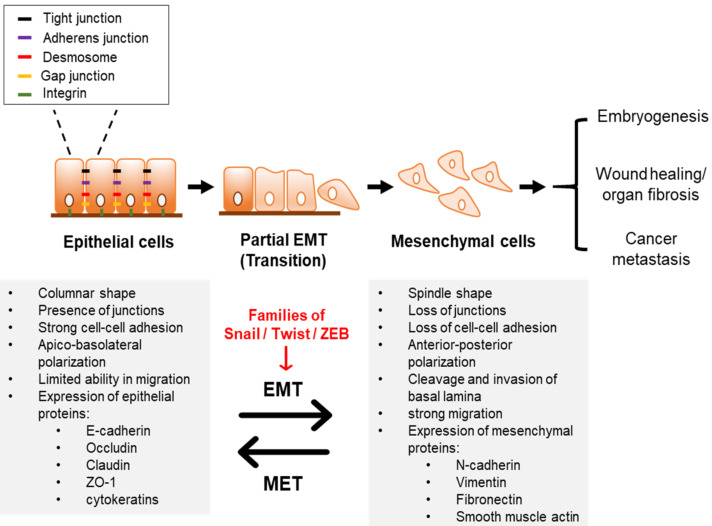
Characteristics of EMT. Epithelial cells are usually attached to the basement membrane. These cells maintain cell–cell connection such as adherens, tight and gap junctions, and desmosomes. EMT is primarily involved in normal embryogenesis and is associated with adult tissue regeneration, wound healing, and fibrosis. As EMT of cancer cells is transient, the mesenchymal state of cells reverts to the epithelial phenotype, which is called as MET. The process of EMT causes epithelial markers (e.g., E-cadherin, claudin, ZO-1) to be gradually lost, while mesenchymal markers (e.g., N-cadherin, vimentin, and fibronectin) to be increased, which causes changes in physiology of cells. Hence, cells acquire high motility and invasive properties. This process is regulated by EMT-TFs such as families of Snail, Twist, and ZEB. EMT, epithelial–mesenchymal transition; EMT-TFs, EMT-inducing transcription factors; MET, mesenchymal–epithelial transition; ZO-1, Zonula occludens-1.

EMT is a natural trans-differentiation program of epithelial cells into mesenchymal cells [2]. EMT is primarily related to normal embryogenesis, including gastrulation, renal development, formation of the neural crest, and heart development [3]. It is also associated with adult tissue regeneration, wound healing, and fibrosis in which tissue fibroblasts originate from endothelial or epithelial cells [4]. Epithelial cells maintain strong apico–basolateral polarity through connections between intercellular junctions, such as adherens, tight, and gap junctions, as well as desmosomes (Figure 1) [5,6]. During EMT, the main characteristics of epithelial cells are gradually lost and undergo partial EMT [2,7,8]. Some of these cells within partial EMT remain cell–cell junction and show both epithelial (e.g., cell–cell adhesion) and mesenchymal (e.g., migration) characteristics [9]. Additionally, endothelial cells have characteristics similar to epithelial cells. Thus, the process of transition from endothelial cells to mesenchymal cells is one of the variants of traditional EMT and is named endothelial-to-mesenchymal transition (EndoMT) [10]. EndoMT is involved in the formation of heart valves and the generation of cancer-associated fibroblasts [11]. Similar to epithelial cells in EMT, endothelial cells also show various intermediate phenotypes in EndoMT [12,13]. Such hybrid cells disseminate as cell clusters [9]. For complete EMT, these hybrid cells then have anteroposterior polarity and separate from each other and from adjacent tissues, acquiring mesenchymal cell characteristics [11,13]. By acquiring mesenchymal-associated phenotypes, the cells gain high migratory and invasive properties [13,14]. These mesenchymal cells enter the circulatory system and invade through the basement membrane and extracellular matrix surrounding the cancer tissue [15]. Interestingly, EMT in cancer cells is transient [16]. Therefore, metastatic cells settle down and proliferate, needing to reverse from mesenchymal to a more epithelial phenotype [17]. This conversion is named as mesenchymal–epithelial transition (MET) [18]. Some studies have proposed that these processes lead to the invasion–metastasis cascade. However, recent studies have questioned the necessity of these transitions in driving metastasis [19]. Therefore, the mechanisms regulating the EMT–MET conversion process still need to be studied.

**Figure 2:**
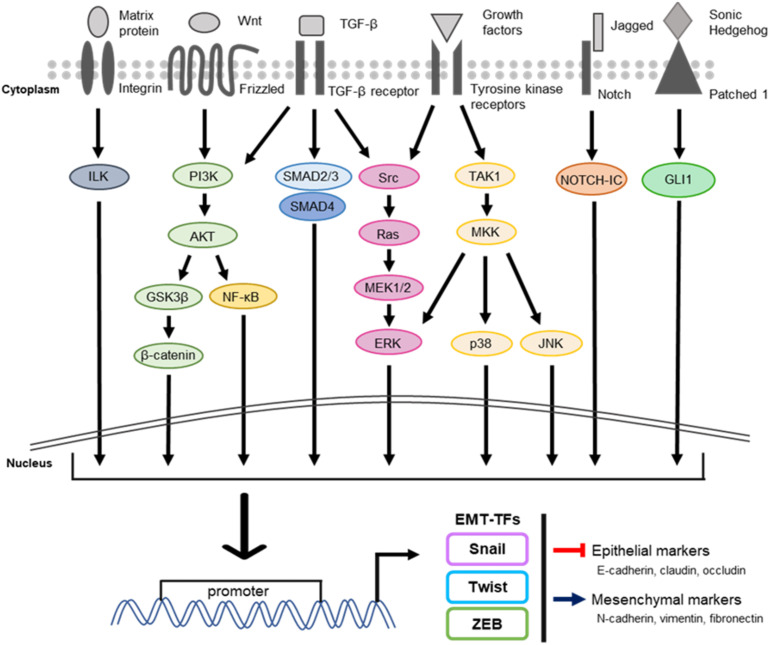
The common signaling pathways regulating EMT. The progression of EMT is controlled by several extracellular and intracellular signaling pathways. Their coordinated interactions bind to DNA promoter regions of EMT-TFs, leading to promotion of transcriptional activity of EMT-TFs. The expressions of EMT-TFs play a key role in regulating the expression of their target genes related to EMT and cancer metastasis. (Left to right: matrix protein/ILK, WNT/PI3K/β-catenin, TGF-β/PI3K/NF-κB, TGF-β/SMAD complex, growth factors or TGF-β/RAS/ERK, growth factors/TAK1/MAPK, Jagged/NOTCH-ICD, and Sonic Hedgehog/GLI1) ILK, integrin-linked kinase; TGF-β, transforming growth factor-beta; PI3K, phosphoinositide 3-kinase; NF-κB, nuclear factor-kappa B; ERK, extracellular signal-regulated kinase; JNK, c-Jun N-terminal kinases; MAPK, mitogen-activated protein kinase; TAK1, transforming growth factor β-activated kinase 1; MEK, MAPK/ERK kinase; MKK, mitogen-activated protein kinase kinase.

In cells, signaling molecules such as hormones, growth factors, and extracellular matrix components bind to specific receptors, which then initiate a physiological response [20]. The intracellular signal transduction is triggered by extracellular signal molecules binding to membrane receptors (Figure 2). Examples of such ligands are growth factors, including epidermal growth factor, fibroblast growth factor, platelet-derived growth factor, transforming growth factor β (TGF-β), bone morphogenetic protein, integrin, Jagged, Wnt, and Sonic Hedgehog [21,22]. These ligands bind to tyrosine receptor kinases, TGF-β receptors, activin receptors, matrix protein, Notch, Frizzled, and patched homolog 1/2 receptor, respectively [7,23]. Many EMT-related signals appear to be cell type- and tissue type-specific [7,24]. Thus, cells could react to signals with different sensitivities or integrate signals differently, depending on the microenvironment and states of cells [7].

**Figure 3:**
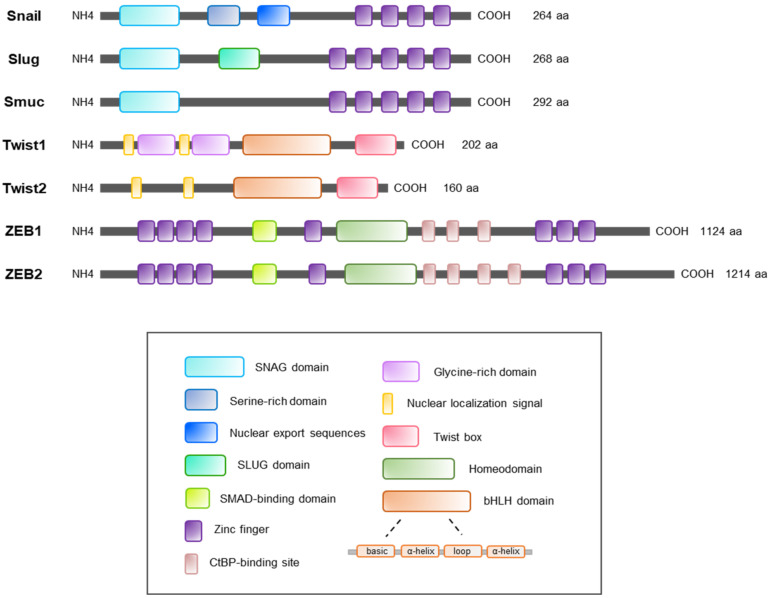
A schematic representation of structures of EMT-TFs. The Snail family (Snail, Slug, and Smuc) commonly includes the SNAG domain in the N-terminal and the zinc finger domain in the C-terminal. Snail contains the nuclear export sequences and serine-rich domain that control the stability of the Snail. The SNAG domain is however only present in Slug. The latest recognized member in Snail family, Smuc does not have both serine-rich domain and SNAG domain. The Twist family (Twist1 and Twist2) mainly consists of a bHLH domain and a Twist box in C-terminal for its transcriptional activity. However, glycine rich domains are only present in Twist1. The bHLH domain consists of basic amino acids followed by two alpha helices, which is separated by a loop of different length. The ZEB family (ZEB1 and ZEB2) has the largest protein size compared to other EMT-TFs. This ZEB family contains various regulatory domains, which include clusters of zinc fingers in N-terminal and C-terminal, homeodomain, SMAD-binding domain, and CtBP-binding site. CtBP, C-terminal binding protein.

Due to the complexity of EMT, molecular and morphological changes in epithelial cells are regulated by activation of specific signaling pathways [25]. Because many signaling pathways are involved in EMT, these pathways interplay with each other and transduce signals through intracellular kinase cascade including phosphoinositide 3-kinase (PI3K)/Akt, mitogen-activated protein kinase (MAPK), SMAD, or nuclear factor-kappa B (NF-κB) [7]. The process of EMT is executed in response to these signaling factors that induce transcription factors (EMT-TFs) such as the families of Snail, Twist, and ZEB, which regulate the expression of EMT-related genes (Figure 3) [26]. Interestingly, all EMT-TFs bind to the E-box motif of the E-cadherin gene promoter region, leading to inhibition of E-cadherin gene expression [27]. During EMT, not only is E-cadherin expression suppressed by EMT-TFs, but they can also downregulate the transcription of other epithelial cell markers such as claudin [27]. On the other hand, it should be noted that EMT-TFs upregulate the markers of mesenchymal cells, including N-cadherin, vimentin, and fibronectin [28,29]. Unfortunately, the underlying mechanisms in which each EMT-TF selectively regulates the expressions of the main EMT-related genes, E-cadherin and N-cadherin, are less-defined. Additionally, the expression of secreted proteases such as matrix metalloproteinases (MMPs) that degrade the extracellular matrix (ECM) around cancer cells, is also upregulated by these EMT-TFs [30]. The physiological roles of EMT-TFs are common in embryogenesis, organism development, and recurrence in cancer cells [31]. As a result, the expressions of EMT-TFs can overlap and form networks [27]. Moreover, EMT-TFs are clinically relevant in metastasis and their expressions are associated with poor outcomes in various cancer patients [32]. Additionally, extensive animal models proved that the overexpression of main EMT-TFs promoted EMT and metastasis [33,34,35]. However, Zheng et al. showed that the loss of Snail and Twist1 gene had no impact on the metastatic spread in pancreatic cancer [36]. In contrast, a recent study of Krebs et al. showed the deletion of ZEB1 gene inhibited metastasis [37]. These studies claim the possibility of partial distribution of EMT-TFs to the mechanisms of cancer metastasis. Therefore, further investigations need to be established to clarify the relationship between EMT-TFs and cancer metastasis.

Even though the transcription of EMT-TFs is regulated by multiple signaling pathways, their protein levels are regulated by post-translational modifications (PTMs) [38]. PTMs are covalent and enzyme-dependent modifications of proteins occurring after protein synthesis to form mature protein product [38,39]. These modifications include the altering the functional group, such as folding or adding of another protein to one or more residues of the target protein [38]. Because the structures of EMT-TFs are different, PTMs have diverse effects on EMT-TFs, an important factor in diversification of protein functions and coordination of their signaling networks [40]. Examples of PTMs include phosphorylation, ubiquitination, sumoylation, acetylation, glycosylation, and methylation [38]. PTMs also function in regulating protein stability, transcriptional activity, and intracellular localization of EMT-TFs [3]. Among these modifications, phosphorylation represents the best-characterized modification involved in various biological activities, such as apoptosis, metabolism, and transcription [41].

Because EMT-TFs are critical factors in regulating EMT-related markers and leading to cancer metastasis, many researchers have studied the signaling pathways and PTMs of EMT-TFs. Furthermore, several studies have revealed that the expression of EMT-TFs induces drug resistance in several malignant carcinomas and leads to poor prognosis for patients [42,43,44]. Therefore, gaining deeper insight into this field may help elucidate the important steps in EMT and cancer metastasis. This review will discuss the current roles and PTM-mediated regulation of EMT-TFs and the functional consequences of these PTMs in cancer metastasis.

## 2. Regulation of the Snail Family

The Snail family consists of the transcription repressor Snail (also referred to as Snail1), Slug (Snail2), and the less characterized Smuc (Snail3), which are located on chromosome loci 20q13.13, 8q11.21, and 16q24.2, respectively [45,46,47,48,49]. This suggests that even through Snail members are in the same family, different gene locations on chromosomes could lead to various gene expressions and regulation. Additionally, Snail and Slug are highly labile proteins with a half-life of about 60 min, due to rapid proteasomal degradation [50,51]. Therefore, understanding of the stabilization and degradation of Snail family proteins is essential in relation to EMT-related gene expression regulation (Table 1).

All members of the Snail family share domains with four to six C2H2 zinc fingers (ZnF) at the DNA-binding C-terminal with high similarity [77,78]. The C-terminal region of Snail family binds to the E-box motif [79]. The central region of Snail contains a nuclear export sequence and a serine-rich domain (SRD), which regulates the stability of Snail protein and its intracellular location [80]. Members of the Snail family contain a SNAG domain, a transactivation domain, in the N-terminal region [81]. The SNAG domain is essential for the binding of transcriptional co-repressors, such as histone deacetylase 1/2 (HDAC 1/2), protein arginine methyl transferase 5, repressor element-1 silencing transcription factor corepressor 1, and polycomb repressive complex 2 [82,83,84,85,86,87]. In addition, the 1 to 9 amino acids of SNAG domain determine the functions of Slug such as the suppression of E-cadherin and induction of EMT [83,88]. Slug also has a unique domain called SLUG, unlike other Snail family members [81,89]. However, Smuc does not contain SRD or SLUG domains, which are main regulatory regions of Snail family.

Snail family has different efficiency in EMT induction [90]. The most well-studied Snail family member, Snail, was first described in *Drosophila melanogaster* [91,92]. Snail binds to promoter regions of target genes with a higher affinity than Slug [93,94]. Furthermore, Snail could be a more potent inhibitor or activator of EMT-related target genes [93,94]. Compared to Snail and Slug, the functions of Smuc in the EMT process of cancer cells are not yet well-known. Nevertheless, recent reports have shown that Smuc is a poor EMT-inducer but involved in cell differentiation [90,95,96].

### 2.1. Snail

#### 2.1.1. Phosphorylation of Snail

The initial phosphorylation of Snail, which is also called “priming”, induces sequential phosphorylation [97]. This priming process is catalyzed by casein kinase 1 at Ser104 and Ser107 [97]. It triggers the following phosphorylation at Ser96 and Ser100 by glycogen synthase kinase 3 beta (GSK3β) [59,97]. These sequential phosphorylation events rely on serine residues of the SRD of Snail protein [98]. When Snail is phosphorylated at Ser96 and Ser100, a well-known E3 ligase called beta-transducin repeat-containing protein (β-TrCP) binds to these phosphorylated sites of Snail [59]. This occurs because β-TrCP has a specific destruction motif, DSGxxS, which is also present in Snail as DSGKSS [50,59]. The main mechanism of regulating Snail stability is by the phosphorylation at specific sites that mediates the E3 ligase binding [59,99].

Considering that these phosphorylated serine residues mediate the binding of E3 ligase to Snail and subsequent ubiquitination, dephosphorylation of these residues would stabilize Snail [63]. The well-known phosphatase of Snail is a small C-terminal domain phosphatase (SCP) family, including SCP1-4, which dephosphorylates GSK3β-phosphorylated residues of Snail [63,64].

p21 activating protein kinase 1 plays a vital role in regulating cell morphogenesis, motility, mitosis, survival, and angiogenesis [100,101]. The phosphorylation of Snail at Ser246 induces nuclear translocation of Snail, which thus activates its transcriptional activity [58]. Large tumor suppressor kinase 2 (Lats2) interacts with Snail in the nucleus and directly phosphorylates Thr203 of Snail [52]. The phosphorylation of Thr203 retains Snail in the nucleus and improves its stability [52]. As a result, Lats2 has a positive effect on EMT and invasion in a Snail-dependent manner [52]. In addition, when protein tyrosine kinase 6 is activated by autophosphorylation at Tyr342, it stabilizes Snail in breast cancer cells [53]. Activation of the extracellular signal-regulated kinase (ERK) by discoidin domain receptor induces phosphorylation of Ser82 and Ser104 of Snail, which in turn causes nuclear accumulation of Snail and leads to the inhibition of E-cadherin expression [57]. One of the central proteins in DNA damage response, ataxia–telangiectasia mutated kinase (referred to as ATM), increases the stability of Snail by phosphorylation at Ser100 [54]. As a result, ATM-mediated phosphorylation of Snail protein induces tumor invasion and metastasis and is also correlated with ionizing irradiation in terms of cellular survival [54,102]. Protein kinase D1 (PKD1) phosphorylates Ser11 of Snail, causing cytoplasmic translocation of Snail from the nucleus through 14-3-3 binding [55]. This process affects the maintenance of the epithelial phenotype of breast cancer cells [103]. Another factor, growth-regulated protein alpha, phosphorylates Ser246 of Snail, which supports the accumulation of Snail in the nucleus and suppresses the expression of E-cadherin [56]. Additionally, casein kinase 2 phosphorylates Ser92 of Snail in vivo and in vitro, and protein kinase A (PKA) phosphorylates Ser11 in vitro [104]. Notably, these kinases stimulate Snail-induced EMT [104].

#### 2.1.2. Ubiquitination of Snail

F-box only protein 11 (FBXO11) is a novel E3 ligase that triggers ubiquitination and subsequent degradation of Snail [60]. FBXO11 needs phosphorylation of Ser11 and binding [60]. With the alanine scanning for substrate recognition, Zheng et al. confirmed that PKD1 phosphorylates Ser11 on the SNAG domain of Snail, which is required for FBXO11 binding [60]. Moreover, F-Box and leucine-rich repeat protein 14 (FBXL14) interacts with Snail and ubiquitinates Lys98, Lys137, and Lys146, independent of GSK3β phosphorylation [61]. Moreover, this study shows that inhibition of FBXL14 via shRNA stabilizes Snail protein [61]. F-box and leucine-rich repeat protein 5 (FBXL5), localized predominantly in the nucleus, interacts with the C-terminal of Snail and polyubiquitinates Lys85, Lys146, and Lys234 of Snail [62]. FBXL5 induces the suppression of Snail protein stability [62].

#### 2.1.3. Acetylation of Snail

CREB-binding protein (CBP), also known as CREBBP, is a histone acetyltransferase (HAT) that functions to add an acetyl group on the lysine residue of a cellular protein [105]. CBP acetylates Lys126 and Lys187 of Snail and consequently enhances Snail target gene expression [65].

#### 2.1.4. Glycosylation of Snail

In the signaling cascade, the *O*-linked β-N-acetylglucosamine (*O*-GlcNAc) modification is the addition of a monosaccharide to especially serine or threonine residues of a target protein [106]. *O*-GlcNAc at Ser112 of Snail could suppress Snail degradation by nuclear translocation and promote EMT [66]. This mode of action suggests that *O*-GlcNAc might suppress phosphorylation at the same residues because phosphorylation occurs reciprocally with *O*-GlcNAcylation [66].

### 2.2. Slug

#### 2.2.1. Phosphorylation of Slug

In the case of Slug, the domains of SNAG and SLUG bind to co-repressors, which are nuclear receptor corepressor and C-terminal binding protein 1 [88]. These co-repressors subsequently stabilize Slug [88]. In particular, the phosphorylation of Slug at Ser4 within the SNAG domain has a modulatory effect on Slug-mediated EMT induction [88]. A recent study by Virtakoivu et al. showed that vimentin directly interacts with ERK and acts as a scaffold to recruit Slug and ERK [67]. This complex regulates the transcriptional activity of Slug by enhancing ERK-mediated Slug phosphorylation at Ser87 [67]. Similar to Snail, Slug is also regulated by the phosphorylation of Ser92, Ser96, Ser100, and Ser104 by GSK3β [48]. Mutation of these residues inhibits degradation of Slug [48]. p21 activating protein kinase 4 promotes prostate cancer progression through direct phosphorylation of Slug at Ser158 and Ser254 [69]. In addition, Slug is temporally mediated by the cyclin E and cyclin-dependent kinase 2 (CDK2) complex during cell cycle [68]. During cell cycle progression, cyclin E generally functions as a regulatory subunit of CDK2, which is essential for G1/S phase progression [68]. This complex phosphorylates Ser54 and Ser104 of Slug in G1/S phase, resulting in ubiquitination and degradation of Slug [68].

#### 2.2.2. Ubiquitination of Slug

The interaction of Slug with FBXL14 promotes degradation of Slug [70]. When all Leu33, Tyr34, Val58, and Trp59 of Slug are mutated to alanine, FBXL14 is unable to interact with Slug [70]. Ubiquitin-specific-processing protease 13 counteracts this activity [107]. Pellino-1, an E3 ligase, activates NF-κB and MAPK signaling pathways in human immune cells [108]. In lung cancer cell lines, overexpressed pellino-1 stabilizes the Slug protein through Lys63-mediated polyubiquitination [74]. Binding β-TrCP and the carboxy terminus of the Hsc70-interacting protein to Slug promotes ubiquitination and subsequent proteasomal degradation of Slug [71,72]. The interaction between these E3 ligases and Slug is affected by GSK3β-mediated phosphorylation [71,72]. The interaction of both p53 and p21 with Slug induces the mouse double minute 2 homolog (MDM2)-mediated degradation of Slug, leading to inhibition of cell invasion [73,109]. For example, in non-small cell lung cancer (NSCLC), high expression of Slug and low expression of E-cadherin and MDM2 are correlated with mutation of p53 gene, which is associated with poor progression [73]. Wang et al. revealed that wild-type p53 not only inhibits Slug gene expression, but also induces MDM2-mediated ubiquitination by forming a complex with MDM2 and Slug [73]. In particular, amino acid residues 21–29 and 27–66 of Slug are essential for interaction with p53 and MDM2 [73].

#### 2.2.3. Sumoylation of Slug

In a mouse model of prostate cancer, p19^Arf^, a mouse homologue of human p14^Arf^, stabilizes Slug and inhibits E-cadherin expression [75]. p14^Arf^, accumulates mainly in the nucleus and generally forms a stable complex with MDM2/p53 [110]. In this process, p14^Arf^ acts as a tumor suppressor, which inhibits p53-dependent cell cycle arrest and apoptosis through MDM2-mediated degradation of p53 [111,112,113]. Interestingly, p14^Arf^ stabilizes Slug by inducing sumoylation at Lys192 of Slug and then inhibits proteasomal degradation of Slug [75].

#### 2.2.4. Acetylation of Slug

Lastly, PTM-mediated regulation of Slug contains acetylation. A deacetylase sirtuin 2 binds directly to Slug and deacetylates Lys116 in the SLUG domain [51]. The overexpression of sirtuin 2 stabilizes Slug by deacetylation in basal-like breast cancer cells (BLBCs) [51]. CBP, which acetylates Snail, also causes acetylation of Slug at Lys166 and Lys211 [76]. CBP-mediated acetylation contributes to the stabilization of Slug and promotes EMT and migration of breast cancer cell lines, MCF-7 and Sum159 [76].

### 2.3. Smuc

Smuc is the most recently emerged member of the Snail family [49]. Many studies have suggested that Smuc has different functional characteristics compared to Snail and Slug [114,115]. Studies on the regulation of Smuc by PTMs are lacking. Revealing the regulation of Smuc by PTMs through continuous research can be a cornerstone for understanding the mechanisms of the Snail family and cancer metastasis.

## 3. Regulation of the Twist Family

The Twist family (Twist1 and Twist2) includes tissue-restricted members that belong to the basic-helix-loop-helix (bHLH) class B family of transcription factor, which was discovered originally in *Drosophila* [116,117]. According to chromosome mapping, human Twist1 and Twist2 genes are mapped on chromosome loci 7p21.2 and 2q37.3, respectively [118].

The Twist family is conserved evolutionarily from *Drosophila* to humans mainly in two regions, the bHLH domain and a Twist box (often called the tryptophan and arginine; WR motif) [119,120]. Structurally, the bHLH motif of the Twist family consists of basic amino acids followed by an amphipathic alpha-helix (first helix), a loop with different lengths, and then another amphipathic alpha-helix (second helix) [120]. Other than the bHLH domain and a Twist box, nuclear localization signals at the N-terminal of the Twist family is present as a functional motif [121]. Human Twist1 and Twist2 share 68% homology and contain almost identical amino-acid sequences in the bHLH domain and Twist box [122]. A region of the Twist box contains amino-acid residues Leu187, Phe191, and Arg195 (LX3FX3R), which has been characterized as both activator and repressor [116]. Even though they share high similarity in structures, major differences exist between Twist1 and Twist2: the size and N-terminal domains of the proteins [116]. The N-terminal of Twist1 contains two glycine-rich regions, not found in Twist2 [116].

Both Twist1 and Twist2 function as molecular switches to activate or suppress target genes directly or indirectly [123]. In mesenchymal cells, the roles of the Twist family in transcriptional regulation of development-related processes have been characterized by genetic studies [122]. Furthermore, the Twist family plays a critical role in inhibition of myogenic and osteoblast maturation and the progression of cancer by facilitating EMT [116,124,125,126]. Many researchers have shown that the expression of Twist1 is associated with poor clinical outcomes and distal metastasis in various solid cancer types such as prostate, cervical, breast, gastric, and pancreatic cancers [124,127,128,129]. Until now, studies on Twist2 have been controversial and thus have required further investigation. For example, the upregulation of Twist2 occurs in various cancer types [130,131]. However, a study by Zhao et al. showed that Twist2 in hepatocellular carcinoma (HCC) displays no effect in invasion and metastasis [132]. Even though few studies have been conducted on PTMs of Twist2, the activity of Twist1 is tightly regulated by PTMs, which offers an alternative to quickly adapt its activity to cellular context (Table 2).

### 3.1. Twist1

#### 3.1.1. Phosphorylation of Twist1

Phosphorylation regulates the activity and stability of Twist1. Phosphorylation at Thr125 and Ser127 by PKA enhances Twist1 dimerization and DNA binding [148]. These phosphorylation sites are suppressed by protein phosphatase 2 [148]. Phosphorylation at Ser68 of Twist1 by MAPK has been reported to increase Twist1 stability in breast cancer cells [133]. The Twist1 S68A mutant results in increased ubiquitination and subsequent degradation of Twist1 [133]. Controlling EMT and invasion in breast cancer cells depends on Twist1 stability [133]. Similar to Snail, SCP1 interacts with and dephosphorylates Ser68 of Twist1, leading to the acceleration of Twist1 degradation and inhibition of cancer invasion [142].

Protein kinase B, also known as Akt, has been suggested to regulate Twist1 stability and activity [149]. A study by Li et al. reported that Akt1 and Akt2 function in a different manner to regulate Twist1 in breast cancer [140]. Akt1 phosphorylates Twist1 at Ser42, Thr121, and Ser123 and induces its degradation via β-TrCP-mediated ubiquitination, which is dependent on pThr121/Ser123 of Twist1 [140]. Akt2 phosphorylates Twist1 only at Ser42, which further results in suppression of Twist1-mediated E-cadherin expression and induces EMT [134,135]. Akt-dependent phosphorylation at Ser42 of Twist1 suppresses p53 activity and triggers cell survival [135]. The phosphorylation level of the Akt family is correlated with Twist1 protein level [150]. In this study, the specificity of Ser42 phosphorylation of Twist1 by Akt is also evaluated in the Akt1/2-deficient mouse embryonic fibroblast [150]. The functions of Twist1 regulated by Akt remain controversial, but Akt1 may play a dual role in the regulation of Twist1 according to the tissue type.

Twist1 expression is upregulated in head and neck squamous cell carcinoma (HNSCC) cell lines treated with interleukin 6 (IL-6). The treatment of IL-6 activates casein kinase 2 alpha which interacts directly with Twist1 and phosphorylates Ser18 and Ser20 of Twist1 [136]. As a result, the stability of Twist1 is enhanced and the motility of HNSCC cells is improved [136]. Protein kinase C alpha (PKCα) also binds to Twist1 via the Twist box domain. Phosphorylation at Ser144 of Twist1 by PKCα reduces ubiquitination and increases stabilization of Twist1 [137]. Aurora A promotes activity of Twist1 by phosphorylating Ser123, Thr148, and Ser184, which is predicted because phosphorylation by Aurora A acts in the direction of inhibiting the ubiquitination of Twist1 [138]. The inhibitor of NF-κB kinase beta induces cytoplasmic translocation of Twist1 for accelerating β-TrCP-mediated destruction by phosphorylating Thr125 and Ser127 of Twist1 [141]. In addition, CD44, a hyaluronan receptor, not only interacts with c-Src and Twist1 in the breast cancer cell line, MDA-MB-231, but also increases tyrosine phosphorylation of Twist1 by activating c-Src kinase, which promotes Twist1 nuclear translocation [139].

#### 3.1.2. Ubiquitination of Twist1

Ubiquitination has not been studied much in the regulation of Twist1 protein stability and activity. FBXL14, previously found to reduce the stability of the Snail family in cancer cells, also regulates the stability of Twist by ubiquitination via C-terminal Twist box [141,143]. Lander et al. demonstrated that the deletion of Twist box leads to a loss of interaction with FBXL14 [143]. Moreover, a depletion of endogenous FBXL14 in embryos shows an increase in Twist1 stability [143].

#### 3.1.3. Acetylation of Twist1

The other well-known PTM of Twist1 is acetylation [151]. Studies have revealed that Twist1 interacts with p300 or p300/CBP-associated factor (PCAF), a well-known HAT and promotes EMT by suppressing the expression of E-cadherin [152] and p53 [153]. PCAF acetylates Twist1 at Lys73, Lys76, and Lys77, which promotes nuclear localization of Twist1 and increases its transcriptional activity in bladder cancer cells [144]. Additionally, another acetyltransferase named Tat-interacting protein of 60 kDa (Tip60), also acetylates Twist1 at Lys73 and Lys76 [145]. Diacetylation of Twist1 at these lysine residues is a prerequisite for the interaction with bromodomain-containing protein 4 [145]. Subsequently, this deacetylation of Twist1 leads to EMT induction and metastasis in HCCs and BLBCs [145,146].

#### 3.1.4. Methylation of Twist1

In general, methylation at arginine of protein via addition of methyl groups can modulate the stability and sub-cellular localization of proteins [154]. This arginine methylation is catalyzed by protein arginine methyl transferases (PRMTs) [154]. PRMTs are upregulated aberrantly in several cancers [155]. For example, protein arginine methyl transferase 1 methylates Arg34 of Twist1, leading to inhibition of E-cadherin expression and cell migration in NSCLC cell line A549 [147]. However, when Arg34 of Twist1 is mutated to lysine, no impact is observed on E-cadherin expression in A549 cells and even in breast cancer cell line MCF7 [147]. Moreover, R34K mutant of Twist1 is located predominantly in the cytoplasm, which suggests that methylation of Twist1 at Arg34 might have a possible role in regulating nuclear translocation of Twist1 [147]. However, the molecular mechanisms of Twist1 translocation by methylation remain to be evaluated.

## 4. Regulation of the ZEB Family

The ZEB family, a family of zinc finger E-box binding homeodomain proteins, consists of two homologous proteins named ZEB1 (also referred as σEF1) and ZEB2 (also known as SIP1). The ZEB family is long-lived relative to other EMT-TFs [27]. The genomes of ZEB1 and ZEB2 are mapped on chromosome loci 10q11.22 and 2q22.3, respectively [156]. According to a study by Vandewalle et al., the similarity of ZEB1 and ZEB2 is shown 43% within humans [157]. However, these two homologs show 89% similarity in other vertebrates [157].

The ZEB family is mainly characterized by the presence of two separated clusters of ZnF domains at both the N- and the C-terminal and the centrally located homeodomain [157]. Two ZnF clusters are known as the most common DNA-binding motif [157]. Interestingly, the N-terminal ZnF (NZF) contains three C2H2 and one CCHC motifs, whereas the C-terminal ZnF (CZF) contains only three CCHC motifs [157]. When ZEB1 and ZEB2 are compared, the sequence identity within NZF and CZF shows a similarity of 88% and 93%, respectively [158,159]. These two clusters of ZEB1 and ZEB2 bind to bipartite E-box-like elements (CACCT) located in many gene promoters, which suggests that both ZEB proteins have similar DNA-binding specificity [157,159]. However, the molecular mechanisms underlying the option between activation and repression of target genes by ZEB1 and ZEB2 are undisclosed. The homeodomain in ZEB1 and ZEB2 protein structure consists of helix-loop-helix motif [157]. The homeodomain does not bind DNA, but mainly participates in protein-protein interaction [160]. Other domains such as a SMAD-binding domain (SBD) and C-terminal binding protein (CtBP)-binding domain are included in the ZEB family [158]. These protein-binding domains are essential in control of transcriptional activity of the ZEB family [161]. Intriguingly, SBD of human ZEB1 and ZEB2 shows noticeably low similarity in structure [159]. This difference may explain why ZEB1 and ZEB2 have different functions and signaling pathways [159]. ZEB1 and ZEB2 often display mirrored expression and effects in tissue homeostasis, tissue differentiation, and development [161]. For instance, a study of melanocyte differentiation showed that ZEB2 is overexpressed in melanoma patients, whereas ZEB1 is not [162]. In addition, osteoblast differentiation is induced by ZEB1, but ZEB2 shows the reverse effects [27].

Similar to other EMT-TFs, several reports have revealed that ZEB1 and ZEB2 are important factors in various malignant cancer types [163,164,165]. Most studies on the regulation of the ZEB family discuss miRNAs, especially the miR-200 family, and cross-regulation of other EMT-TFs [166,167]. However, the PTM-mediated regulation of the ZEB family in EMT is not studied extensively (Table 3).

### 4.1. ZEB1 and ZEB2

#### 4.1.1. Phosphorylation of ZEB1 and ZEB2

Phosphorylation mainly modifies the ability of ZEB1 to interact with several coactivators or corepressors for regulating its transcriptional activity [180]. Binding of insulin-like growth factor-1 (IGF-1) to its receptor causes activation of tyrosine kinase, which activates multiple signaling pathways including downstream MAPK [181]. Treatment of IGF-1 not only reduces transcriptional activity of ZEB1 through phosphorylation of Thr851, Ser852, and Ser853 by protein kinase C (PKC), but also activates ERK, resulting in phosphorylation of Thr867 in ZEB1 [170]. Phosphorylation of ZEB1 through PKC-mediated ERK activation prevents nuclear accumulation of ZEB1, thereby reducing its transcriptional activity [170]. According to Jhang et al., ATM phosphorylates ZEB1 at Ser585 and stabilizes ZEB1, which subsequently interacts with ubiquitin-specific-processing protease 7, inducing radiation resistance [168,169]. The phosphorylation-mediated mechanism of ZEB2 is poorly known, but a recent study has shown that the residues between Ser705 and Tyr802 are phosphorylated by GSK3β, reducing the stability of ZEB2 [178].

#### 4.1.2. Ubiquitination of ZEB1 and ZEB2

One of the well-known F-box proteins associated with EMT-TFs, FBXL14 interacts with ZEB2 and leads to ubiquitination-mediated degradation [143]. However, the detailed mechanism is not known [143]. F-box only protein 45 (FBXO45), as a substrate-recognition subunit of E3 ubiquitin ligase, forms a complex with S-phase kinase-associated protein 1 and myc-binding protein 2 [179]. The F-box domain of FBXO45 interacts with Lys48 of ZEB2, which promotes its degradation [179]. Intriguingly, the F-box/WD repeat-containing protein 7 (FBXW7) causes ubiquitination of ZEB2 in a GSK3β-dependent phosphorylation manner. Subsequently, FBXW7 mediates proteasomal degradation of ZEB2 [178]. Because ubiquitination is a reversible process, ubiquitin chains are removed by deubiquitinating enzymes (DUBs) [182]. Among DUBs, ubiquitin specific peptidase 51 (USP51) binds to the N-terminal of ZEB1 and increases ZEB1 protein stability in breast cancer cell lines [171]. Consequently, USP51 upregulates the mesenchymal markers including N-cadherin and vimentin [171]. Furthermore, another DUB, COP9 signalosome subunit 5 (CSN5), is found in various cancers such as colorectal cancer [183]. CSN5 deubiquitinates ZEB1 by interacting with it directly, which increases ZEB1 stability [172,183].

#### 4.1.3. Sumoylation of ZEB1 and ZEB2

Sumoylation of ZEB1 and ZEB2 induces EMT by inhibiting E-cadherin expression [173]. Polycomb protein 2, named Pc2, acts as a small ubiquitin-related modifier E3 ligase, and causes sumoylation by binding to ZEB1 and ZEB2 [173]. Pc2 sumoylates Lys347 and Lys774 of ZEB1 and Lys391 and Lys866 of ZEB2 [173]. According to a study by Long et al., the sumoylation at Lys866 of ZEB2 interferes with the interaction with CtBP, leading to up-regulation of E-cadherin expression [173].

#### 4.1.4. Acetylation of ZEB1 and ZEB2

When p300 and PCAF are bound to the N-terminal domain of ZEB1, Lys741, Lys774, and Lys775 of ZEB1 are acetylated [174]. Acetylated ZEB1 reduces the binding affinity to CtBP, thereby increasing its transcription activity [184]. Tip60 interacts with the N-terminal of ZEB1, which represses the activity of ZEB1 protein [175]. The repressive mechanisms of ZEB1 by Tip60 is an ongoing investigation. Nucleosome remodeling and deacetylation (NuRD) complexes contains HDAC1/2 and chromodomain helicase DNA-binding proteins [185]. The NuRD complex binds to both ZEB1 and ZEB2 [186]. This complex binds to the NuRD-interacting motif that is close to the N-terminal of ZEB2 [186]. The domains of ZEB1 that interact with the NuRD complex are not known [187]. Interestingly, HDAC1/2 interacts with ZEB1 and ZEB2 [176,177,188]. The HDAC1/2 and ZEB1 complex induces the suppression of E-cadherin expression [176,177,188]. Even though the research of Wu et al. has shown that HDAC1/2 interacts with ZEB2 through binding to Arg22, this interaction reportedly affects only the differentiation of Schwann cells during myelination [188]. Therefore, studies on EMT induction by the interaction of HDAC1/2 and ZEB2 still need to be investigated [188].

Recently, a number of studies on structure and mechanisms of the ZEB family have been reported. Within this research, the PTM-mediated regulation of ZEB stability and activity has been studied actively, but not as deeply as the PTM-mediated regulation of the Snail and Twist families. The ZEB family of EMT-TFs not only plays a role in inducing cancer metastasis, but also promotes cancer stem cell-like properties in various cancers [161,189].

## 5. Concluding Remarks

The different expressions of the core EMT-TFs, namely the Snail, Twist, and ZEB families are observed in development, tissue homeostasis, and carcinogenesis and are modulated by several intracellular signaling pathways. These EMT-TFs are also associated with cancer drug resistance through various molecular mechanisms. Recently, studies on PTM-mediated regulation of EMT-TFs in various cancers have been investigated and developed more deeply. This review highlights the mechanisms of PTMs associated with EMT-TFs. Critical regulators of PTMs on EMT-TFs have been studied, but more investigations on the mechanisms are needed. Additionally, a better understanding of the mechanisms underlying the relationship between drug resistance and PTMs is necessary. Ultimately, a thorough understanding of EMT-TFs from the perspective of PTMs will pave the way for overcoming cancer metastasis by developing therapeutic approaches that can modulate PTMs of EMT-TFs.

## Figures and Tables

**Table 1:** Regulation of the Snail family by post-translational modifications (PTMs).

Transcription Factor	Function	Effects on EMT-TFs	Regulation Factor	Role or Mechanism	Reference
Snail	Phosphorylation	Stabilization	Lats2	Phosphorylation at Thr203	[52]
			PTK6	Phosphorylation at Tyr342	[53]
			ATM	Phosphorylation at Ser100	[54]
		Degradation	PKD1	Phosphorylation at Ser11	[55]
		Nuclear accumulation	GROα	Phosphorylation at Ser246	[56]
			ERK	Phosphorylation at Ser82 and Ser104	[57]
			PAK1	Phosphorylation at Ser246	[58]
	Ubiquitination	Degradation	β-TrCP	Ubiquitination at pSer96 and pSer100	[59]
			FBXO11	Ubiquitination at pSer11	[60]
			FBXL14	Ubiquitination at Lys98, Lys137, and Lys146	[61]
			FBXL5	Ubiquitination at Lys85, Lys146, and Lys234	[62]
	Dephosphorylation	Stabilization	SCP	Dephosphorylation at Ser96 and Ser100	[63,64]
	Acetylation	Increasing transcriptional activity	CBP	Acetylation at Lys126 and Lys187	[65]
	Glycosylation	Stabilization	*O*-GlcNAc	*O*-GlcNAc at Ser112	[66]
Slug	Phosphorylation	Nuclear translocation	ERK-vimentin complex	Phosphorylation at Ser87	[67]
		Degradation	GSK3β	Phosphorylation at Ser92, Ser96, Ser100, and Ser104	[48]
			CDK2	Phosphorylation at Ser54 and Ser104	[68]
		Stabilization	PAK4	Phosphorylation at Ser158 and Ser254	[69]
	Ubiquitination	Degradation	FBXL14	Ubiquitination depends on Leu33, Tyr34, Val58, and Trp59	[70]
			β-TrCP	GSK3β-mediated ubiquitination	[71]
			CHIP	GSK3β-mediated ubiquitination	[72]
			p53	MDM2-mediated ubiquitination	[73]
		Stabilization	pellino-1	Ubiquitination at Lys63	[74]
	Sumoylation	Stabilization	p14^Arf^	Sumoylation at Lys192	[75]
	Acetylation	Stabilization	Sirtuin 2	Deacetylation at Lys116	[51]
			CBP	Acetylation at Lys166 and Lys211	[76]

Lats2, large tumor suppressor kinase 2; PTK6, protein tyrosine kinase 6; ATM, ataxia–telangiectasia mutated kinase; PKD1, protein kinase D1; GROα, growth-regulated protein alpha; ERK, extracellular signal-regulated kinase; PAK1, p21 activating protein kinase 1; β-TrCP, beta-transducin repeat-containing protein; FBXO11, F-box only protein 11; FBXL14, F-Box and leucine-rich repeat protein 14; FBXL5, F-box and leucine-rich repeat protein 5; SCP, small C-terminal domain phosphatase; CBP, CREB-binding protein; O-GlcNAc, O-linked β-*N*-acetylglucosamine; GSK3β, glycogen synthase kinase 3 beta; CDK2, cyclin-dependent kinase 2; PAK4, p21 activating protein kinase 4; CHIP, carboxy terminus of the Hsc70-interacting protein.

**Table 2:** Regulation of the Twist family by PTMs.

Transcription Factor	Function	Effects on EMT-TFs	Regulation Factor	Role or Mechanism	Reference
Twist1	Phosphorylation	Stabilization	MAPK	Phosphorylation at Ser68	[133]
			Akt2	Phosphorylation at Ser42	[134,135]
			CK2α	Phosphorylation at Ser18 and Ser20	[136]
			PKCα	Phosphorylation at Ser144	[137]
			Aurora A	Phosphorylation at Ser123, Thr148, and Ser184	[138]
		Nuclear translocation	CD44	c-Src-dependent phosphorylation at tyrosine	[139]
		Degradation	Akt1	Phosphorylation at Ser42, Thr121, and Ser123	[140]
			IKKβ	Phosphorylation at Thr125 and Ser127	[141]
	Dephosphorylation	Degradation	SCP1	Dephosphorylation at Ser68	[142]
	Ubiquitination	Degradation	β-TrCP	Ubiquitination at Thr121 and Ser123	[140]
			FBXL14	C-terminal Twist box-dependent ubiquitination	[143]
	Acetylation	Nuclear translocation	PCAF	Acetylation at Lys73, Lys76, and Lys77	[144]
		Nuclear translocation	Tip60	Acetylation at Lys73 and Lys76	[145,146]
	Methylation		PRMT1	Methylation at Arg34	[147]

MAPK, mitogen-activated protein kinase; CK2α, casein kinase 2 alpha; PKCα, protein kinase C alpha; IKKβ, inhibitor of NF-κB kinase beta; SCP1, small C-terminal domain phosphatase 1; β-TrCP, beta-transducin repeat-containing protein; FBXL14, F-Box and leucine-rich repeat protein 14; PCAF, p300/CBP-associated factor; Tip60, tat-interacting protein of 60 kDa; PRMT1, protein arginine methyl transferase 1.

**Table 3:** Regulation of the ZEB family by PTMs.

Transcription Factor	Function	Effects on EMT-TFs	Regulation Factor	Role or Mechanism	Reference
ZEB1	Phosphorylation	Stabilization	ATM	Phosphorylation at Ser585	[168,169]
		Inhibition of transcriptional activity	PKC	Phosphorylation at Thr851, Ser852, and Ser853	[170]
			ERK	Phosphorylation at Thr867	[170]
	Deubiquitination	Stabilization	USP51	Binding to N-terminal of ZEB1	[171]
			CSN5	Binding to ZEB1	[172]
	Sumoylation	Inhibition of transcriptional activity	Pc2	Sumoylation at Lys347 and Lys774	[173]
	Acetylation	Inhibition of transcriptional activity	p300 and PCAF	Acetylation at Lys741, Lys774, and Lys775	[174]
			Tip60	Binding to N-terminal of ZEB1	[175]
	Deacetylation	Increasing transcriptional activity	HDAC1/2	Binding to ZEB1	[176,177]
ZEB2	Phosphorylation	Degradation	GSK3β	Phosphorylation at Ser705 and Tyr802	[178]
	Ubiquitination	Degradation	FBXL14	Binding to ZEB2	[143]
			FBXO45	Ubiquitination at Lys48	[179]
			FBXW7	GSK3β-mediated ubiquitination	[178]
	Sumoylation	Inhibition of transcriptional activity	Pc2	Sumoylation at Lys391 and Lys866	[173]

ATM, ataxia–telangiectasia mutated kinase; PKC, protein kinase C; ERK, extracellular signal-regulated kinase; USP51, ubiquitin-specific peptidase 51; CSN5, COP9 signalosome subunit 5; Pc2, polycomb protein 2; PCAF, p300/CBP-associated factor; TIP60, tat-interacting protein of 60 kDa; HDAC1/2, histone deacetylase 1/2; GSK3β, glycogen synthase kinase 3 beta; FBXL14, F-Box and leucine-rich repeat protein 14; FBXO45, F-box only protein 45; FBXW7, F-box/WD repeat-containing protein 7.

## Data Availability

Not applicable.

## Abbreviations

ATM	Ataxia–telangiectasia mutated kinase
BLBCs	Basal-like breast cancer cells
β-TrCP	Beta-transducin repeat-containing protein
CBP	CREB-binding protein
CDK2	Cyclin-dependent kinase 2
CSN5	COP9 signalosome subunit 5
CtBP	C-terminal binding protein
CZF	C-terminal zinc finger
DUBs	Deubiquitinating enzymes
ECM	Extracellular matrix
EMT	Epithelial–mesenchymal transition
EMT-TFs	EMT-transcription factors
EndoMT	Endothelial-to-mesenchymal transition
ERK	Extracellular signal-regulated kinases
FBXL14	F-Box and leucine-rich repeat protein 14
FBXL5	F-box and leucine-rich repeat protein 5
FBXO11	F-box only protein 11
FBXO45	F-box only protein 45
FBXW7	F-box/WD repeat-containing protein 7
GSK3β	Glycogen synthase kinase 3
HAT	Histone acetyltransferase
HCC	Hepatocellular carcinoma
HDAC1/2	Histone deacetylase 1/2
HNSCC	Head and neck squamous cell carcinoma
IGF-1	Insulin-like growth factor-1
IL-6	Interleukin 6
Lats2	Large tumor suppressor kinase 2
MAPK	Mitogen-activated protein kinase
MDM2	Mouse double minute 2 homolog
MET	Mesenchymal–epithelial transition
MMPs	Matrix metalloproteinases
NF-κB	Nuclear factor-kappa B
NSCLC	Non-small cell lung cancer
NuRD	Nucleosome remodeling and deacetylation
NZF	N-terminal zinc finger
O-GlcNAc	O-linked β-*N*-acetylglucosamine
Pc2	Polycomb protein 2
PCAF	p300/CBP-associated factor
PI3K	Phosphoinositide 3-kinase
PKA	Protein kinase A
PKC	Protein kinase C
PKCα	Protein kinase C alpha
PKD1	Protein kinase D1
PRMTs	Protein arginine methyl transferases
PTM	Post-translational modification
SBD	SMAD-binding domain
SCP	Small C-terminal domain phosphatase
SRD	Serine-rich domain
TGF-β	Transforming growth factor beta
Tip60	Tat-interacting protein of 60 kDa
USP51	Ubiquitin specific peptidase 51
ZnF	Zinc finger
